# Comparative Recovery of Serratia marcescens Using Bags versus Gloves as Described in ASTM E1174-21 Health Care Personnel Handwash Method

**DOI:** 10.1128/spectrum.01288-23

**Published:** 2023-05-18

**Authors:** Elizabeth Moyer, Gregory Cole, Eleanor Harding, Marilena Jamieson-Popp, Janice L. Fuls

**Affiliations:** a Henkel Corporation, Stamford, Connecticut, USA; University of Cincinnati

**Keywords:** antibacterial, antimicrobial efficacy, antiseptic, clinical methods, handwashing, health care personnel hand wash

## Abstract

The ASTM E1174-21 Health Care Personnel Handwash method is prescribed by the U.S. Food and Drug Administration (FDA) to demonstrate the efficacy of antiseptic handwashing products. The standardized method allows for marker bacteria to be collected from the hands by using either a bag or a glove. Two recent studies utilizing the different collection methods testing the same product showed substantial differences in results. We sponsored two independent studies to compare the bag and glove collection methods following contamination with Serratia marcescens. Overall, there was no difference between collection methods for bacteria recovered (*P = *0.603). The distribution of recovery for the bag method was slightly less variable than for the glove method. Statistical differences were observed within each lab based on the collection day. The day-to-day variability is critical to consider for future multiple-day studies. Additionally, hand size appears to impact recovery, especially for the glove method, with both small and medium hand sizes resulting in higher recovery than large and extralarge hand sizes (*P = *0.015), whereas hand size did not impact recovery with the bag method (*P = *0.315). While it appears that both the bag and glove methods can be used, our findings suggest that gloves may not be the best option for subjects with large to extra-large hands. Additional work looking at bacterial recovery following product treatment is warranted to understand the impact of large hands in the bag versus glove recovery method.

**IMPORTANCE** Antiseptic hand wash products are evaluated using the standard ASTM E1174-21 to demonstrate their antibacterial efficacy. Often products are tested at multiple labs, and the need to understand variables that may play a role in the outcome of the study is important. This work allows us to evaluate the impact that the two collection methods, bags and gloves, have on bacteria recovery. If differences are observed, standardization to one method may be critical to ensure similar test results when planning studies at multiple labs.

## INTRODUCTION

In 2017, the U.S. Food and Drug Administration (FDA) issued a final monograph for antiseptic ingredients used in a health care setting. In this monograph, the FDA prescribed the use of the American Society for Testing and Materials (ASTM) E1174-13 Health Care Personnel Handwash method to demonstrate the efficacy of antiseptic ingredients used in handwashing agents (1, 2). This method was originally established as an ASTM standard test method in 1987 and was referenced in the 1994 Tentative Final Monograph for Health-Care Antiseptic Drug Products (3). E1174 has gone through several revisions since 1987. The standard has been revised an additional five times, with the latest revision being published in 2021 (4). Some examples of the revisions made over the years include the addition of Escherichia coli as an alternative test organism, changes to inoculum ranges, and removal of multiple washes to reflect the current FDA requirement of only a single wash. The most recent revision to E1174-21 added a precision and accuracy statement. In 2021, the ASTM subcommittee E35-2001 completed an interlaboratory statistical analysis on studies run at multiple labs using a single test product (5). The analysis determined the greatest introduction of variability at baseline was attributed to subjects (44%), hands for the same subject (18%), laboratory (22%), and studies run within a single lab (15%) (5).

All research methods have inherent variability that must be understood, i.e., independent, dependent, and extraneous variables (6). For instance, in E1174, independent variables such as wash time and number of washes can be varied to measure the effect on log_10_ reduction. Factors contributing to the effectiveness of both soaps and the methods to test them have been previously considered and summarized (7–9). Variability has been mostly attributed to extraneous variables such as subjects, different labs, and hands.

FDA’s new requirement to support the efficacy of the antiseptic ingredients is for two independent laboratories to test the activity of the antiseptic ingredient (1). To meet this requirement, studies were performed on an antiseptic product at two independent labs. The results were significantly different. As a result, we wanted to further examine E1174 and determine what differences in execution may have led to the differing results. In reviewing the steps outlined in the standard, it was determined that the two labs differed in how the bacteria were recovered from the hands. E1174 allows for either the use of plastic bags or gloves for recovery. Upon review of the historical versions of E1174, both bags and gloves were found to be a part of the original 1987 version. While we cannot pinpoint historically why both bags and gloves were allowed, we deduct, from the reference found in the ASTM standard, that the use of gloves came from the evaluation of surgical scrubs (5). Published studies demonstrating equivalency between bags and gloves cannot be found. The objective of this study was to determine if bacterial sampling collection using bags was equivalent to the bacterial sampling collection using gloves following contamination with Serratia marcescens as part of the Health Care Personnel Handwash method. Two independent laboratories, Henkel Research@Elm (Henkel), and SGS Stephens, Inc. (SGS), executed these studies comparing the bag and glove methods.

## RESULTS AND DISCUSSION

### Bag versus glove variability.

**FIG 1:**
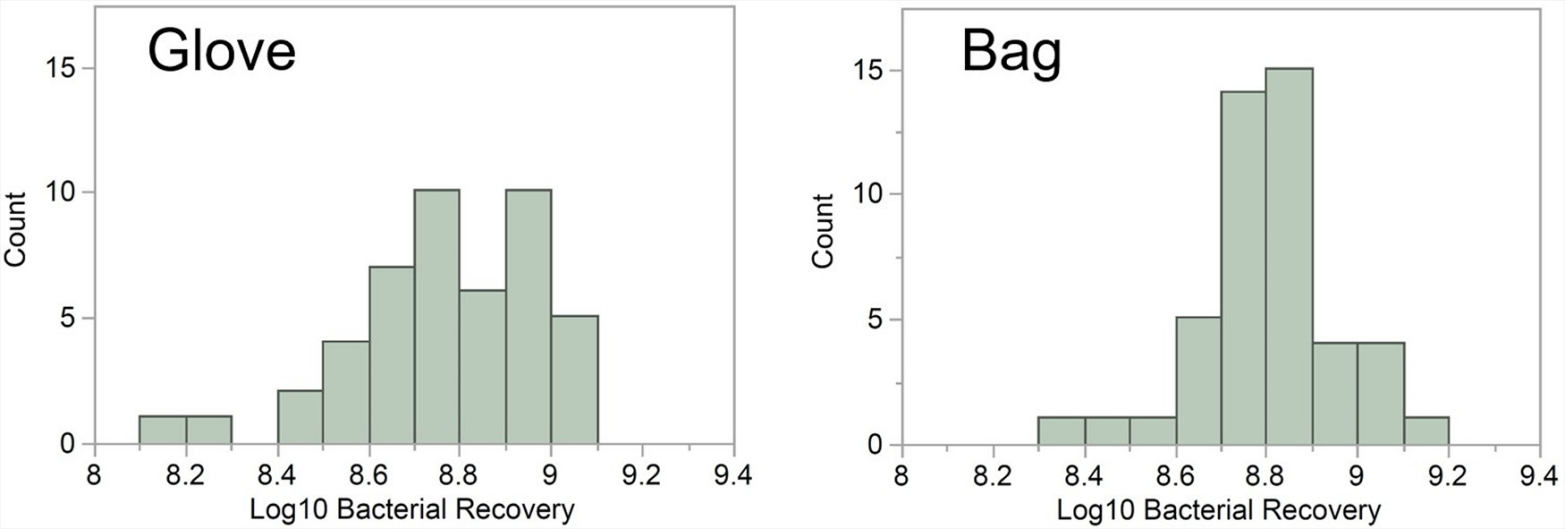
Log_10_ bacterial recovery distribution by collection method. The bag method had a normal distribution and was slightly less variable, having a standard deviation of 0.15. The glove method was nonnormally distributed with a standard deviation of 0.21.

There was not a statistically significant difference in the bacterial counts from samples collected using the bag method (average log_10_ recovery, 8.81) and samples collected using the glove method (average log_10_ recovery, 8.77) (*P = *0.603) (Table 1). The distribution of data for the bag method was slightly less variable, having a standard deviation of 0.15 compared to a standard deviation of 0.21 for the glove method (Fig. 1).

**TABLE 1:** Log_10_ bacterial recovery means by collection method at two independent laboratories^*b*^

Lab	No. of samples	Log_10_ bacterial recovery for:		Log_10_ difference^*a*^
		Bags^*a*^	Gloves^*a*^	
Henkel	24	8.81 ± 0.11	8.78 ± 0.22	0.04 ± 0.25
SGS	22	8.80 ± 0.19	8.76 ± 0.20	0.04 ± 0.26
All data	46	8.81 ± 0.15	8.77 ± 0.21	0.04 ± 0.25

a Values are means ± standard deviations.

b While no significant differences were observed between methods by either lab, the bag method trended higher log_10_ recovery than the glove method.

### Collection day impact on bacterial recovery.

**FIG 2:**
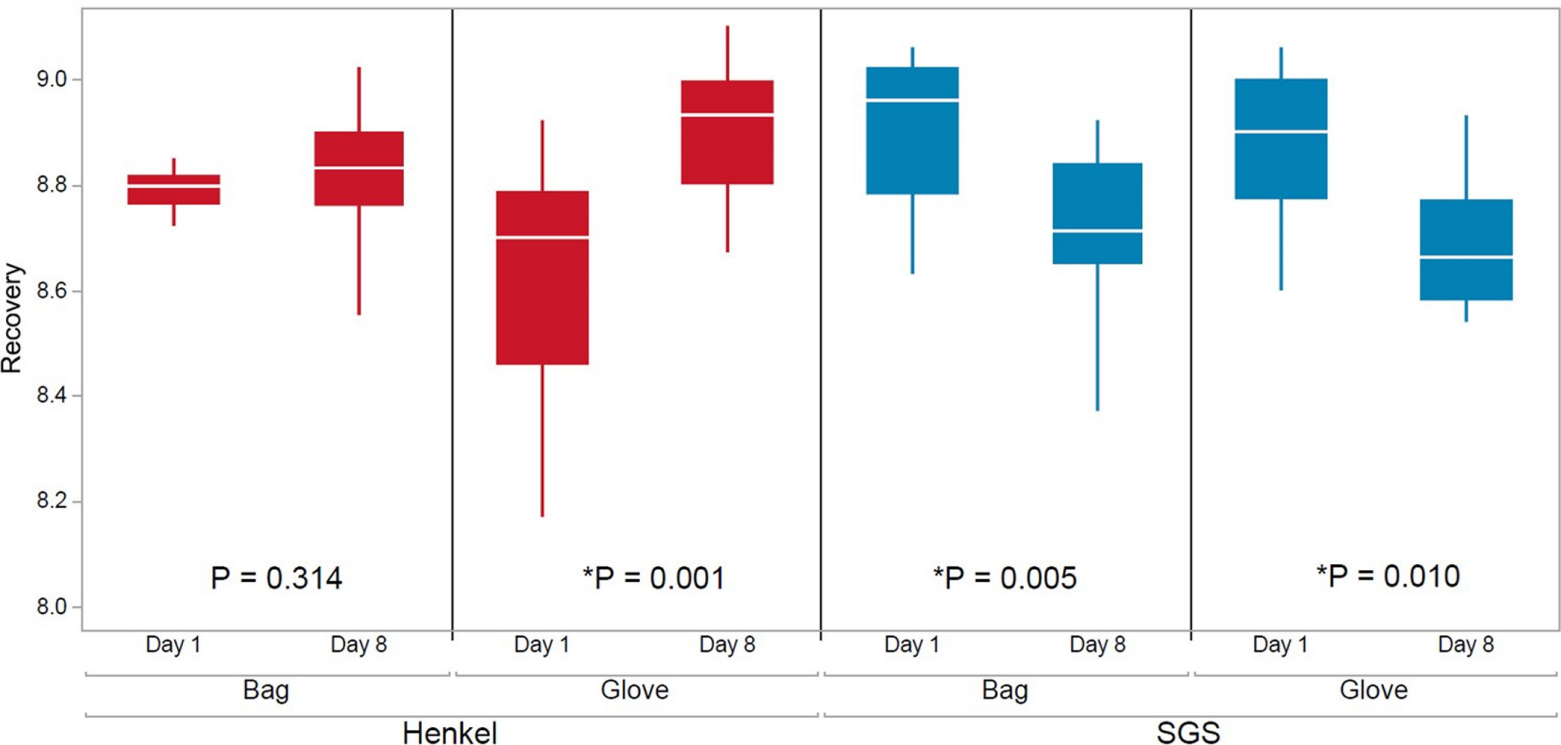
Log_10_ bacterial recovery means by study collection day. Differences were seen by day with both collection methods and with both labs. *, *P* is statistically significant at a 95% confidence level.

There was a statistically significant difference in bacterial counts from the first bacterial collection (day 1) to the second bacterial collection (day 8) at both independent laboratories. For the bag collection method, SGS had observed a 0.21-log_10_ recovery difference between days (*P = *0.005), while Henkel saw a 0.05-log_10_ difference (*P = *0.314). With the glove collection method, both sites had a significant difference between days, with Henkel having a 0.27-log_10_ difference (*P = *0.001), while SGS had a 0.20-log_10_ difference (*P = *0.010) (Fig. 2). Day-to-day variability was observed at both labs, which could be especially critical to consider for multiple-day studies, such as pivotal studies where data can be collected over multiple days or weeks.

### Hand size impact on bacterial recovery.

**FIG 3:**
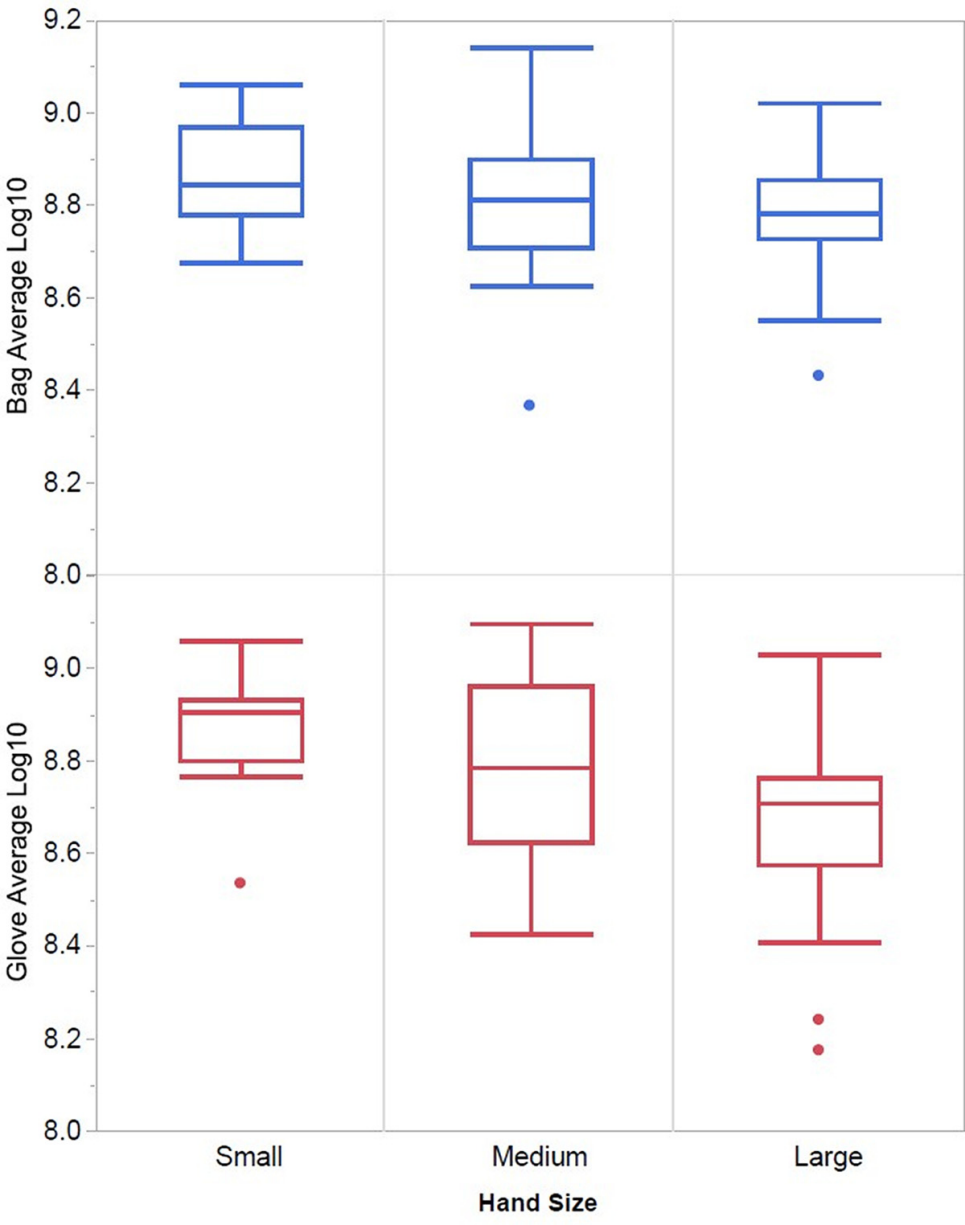
Log_10_ bacterial recovery by surgical glove size and collection method. With the bag method, there was no difference in means between the three hand sizes (*P* = 0.316). With the glove method, there was statistically less recovery for large/extralarge hand sizes than for small and medium hands (*P* = 0.015).

Bacterial recovery was highest for subjects with small hands and decreased with medium and large hands for both collection methods (Fig. 3). When using the glove collection method, there was a statistically significant effect of hand size, with small hands recovering 8.88 log_10_, medium hand sizes recovering 8.82 log_10_, and large/extra-large hand sizes recovering 8.54 log_10_ (*P = *0.015). There was not a statistically significant effect on bacterial recovery based on hand size when using the bag collection method, with recoveries ranging between 8.84 log_10_, 8.82 log_10_, and 8.75 log_10_, respectively (*P = *0.315). Due to the inherent variability within plate count methods, two counts are considered different if the difference is >0.3 log_10_ (10). Only with the glove method was there a difference greater than 0.3 log_10_ between hand sizes.

### Sex, race, and age impact on bacterial recovery.

The data were evaluated by demographics, including sex, race, and age grouping. For sex, there was a statistically significant difference in bacterial recovery, with the glove method observing a higher log_10_ recovery of 8.76 in female participants, while males had a log_10_ recovery of 8.54 (*P = *0.001), which correlates with males typically having larger hands. There was no statistical difference utilizing the bag method for sex (*P = *0.604). Race and age categories did not impact bacterial recovery for either method.

### Further implications.

Understanding the potential sources of variation within a standardized method is critical in its successful execution and interpretation of results. Since the FDA requires testing at multiple sites to confirm the antiseptic activity of a test article, there is higher potential for variability in results.

It is important to note that these studies only examined baseline recovery. Additional differences may be observed when a test product application is examined. With the full E1174 test method, the need for adequate neutralization and ensuring the hands are covered with the stripping solution during massaging is critical to reliable results. In addition, bags are less expensive and do not need to be tested for inhibitory properties as required for the gloves to be used. Additional work looking at bacterial recovery following product treatment is warranted to understand the impact of large hands in the bag versus glove recovery methods. The surgical gloves used in these studies are extralarge for all subjects; however, it is often observed that it is difficult to don large and extralarge subjects’ hands. The hands are typically wet with either bacteria or product, and the insertion into the glove becomes difficult. The data indicate that gloves may not be the best option for subjects with large to extralarge hands. The bag recovery may be better, as there were no recovery differences between hand size or sex.

## MATERIALS AND METHODS

### Subjects.

An independent institutional review board approved both protocols involving human subjects, and all research complied with all federal and institutional guidelines. Healthy adult subjects between 18 and 65 years of age with hands and wrists free of dermatoses, cuts, lesions, and other skin disorders were recruited to participate in the studies. A total of 46 subjects were enrolled in and completed both studies (24 subjects at Henkel and 22 at SGS).

### Study design.

The studies were conducted in the spring of 2022 and utilized a randomized, single-blinded, crossover design. Subjects were randomly assigned to one treatment group (bags or gloves) for the first study visit and the opposite treatment group for the second visit. Each site followed the same protocol. After informed consent was obtained, subjects participated in a 7-day washout during which they were required to refrain from the use of antibacterial products. Subjects were provided a washout kit containing nonantibacterial soaps, alcohol-based hand sanitizer, and gloves for home cleaning. The alcohol-based hand sanitizer was included in this protocol due to the COVID-19 pandemic at the time of study execution and is not known to be persistent.

After completion of the washout, subjects visited the study site to confirm any changes in health status, and their hands were evaluated for cuts, scratches, or issues that would preclude participation. The hand size of participants was measured according to a standard surgical glove sizing chart, which measured the distance from the thumb bridge to the outer aspect of the hand and classified it to small (7 in.), medium, (8 in.), large (9 in.), or extralarge (10 in.). Once enrolled, bacterial sampling was performed in two ways, collection in plastic bags (bag method) and collection in sterile disposable surgical gloves (glove method). With both methods, two laboratory technicians, known as hand massagers, were assigned to one subject. One massager was responsible for the right hand of the subject, and one massager was responsible for the left hand of the subject.

The same massagers were assigned to a subject and performed both sampling procedures. Subjects were randomized so that half of the subjects had the bacterial sampling collected from the bag method, and half of the subjects had the bacterial sampling collected from the glove method. All subjects then participated in a second 7-day washout prior to returning to the study site for the final visit and bacterial sampling (day 8). At the final visit, the subjects had the bacterial sampling collected by the method not performed during test wash 1. Subjects remained in the study for 2 days as a follow-up period to monitor their hands for signs of skin infection.

### Microorganisms and growth conditions.

The bacterial strain used in these studies was Serratia marcescens (ATCC 14756). It was obtained from American Type Culture Collection (ATCC) and was propagated according to ATCC recommendations. Stock cultures were maintained using Microbank bacterial preservation system (Pro-Lab, Diagnostics, Austin, TX) and stored at 80°C. The organism was grown in tryptic soy broth (TSB) (Becton, Dickinson, and Company, Sparks, MD) at 20 to 25°C for 24 ± 2 h. A 24-h broth culture was streaked onto Trypticase soy agar (TSA) (Becton, Dickinson, and Company, Sparks, MD) and incubated for 24 ± 2 h at 20 to 25°C. A study challenge pool was made by transferring at least three isolated colonies from the TSA plate to a sterile vessel of TSB. A series of at least two, but no more than five, 24-h broth transfers were made in 10 mL of TSB. To achieve the appropriate volume of inoculum, TSB was inoculated with 0.1 mL of culture per 100 mL of broth and incubated at 20 to 25°C for 24 ± 4 h. This inoculum was used without dilution in the studies at a titer between 5.0 × 10^8^ CFU/mL and 4.0 × 10^9^ CFU/mL.

### Conditioning wash.

A conditioning wash was performed prior to the start of each test to remove any dirt and oil present on the hands. Subjects were asked to pass their hands under running tap water tempered to 40 ± 2°C. Two pumps of nonantimicrobial soap (Johnson & Johnson Head-to-Toe Wash & Shampoo; Skillman, NJ) were dispensed into the cupped palm of one hand. The soap was spread over the entire surface of the hands and the lower third of the forearms. Subjects washed for 15 ± 2 s following handwashing guidelines from the WHO, making sure all surfaces of the hands, fingers, and backs of hands were washed (11). The hands were rinsed under running tap water tempered to 40 ± 2°C for 30 s. The subjects then dried their hands thoroughly using disposable paper towels. After drying, the hands and wrists were soaked with 70% isopropyl alcohol for 30 s. The hands were then air-dried completely.

### Bacterial contamination.

Subjects’ hands were contaminated with the marker organism following the procedure outlined in ASTM E1174. Three separate 1.5-mL aliquots were placed into the hands and rubbed by the subjects for 20 s. Between each aliquot, the hands dried for 30 s, with the final drying being 90 s prior to recovery. The hands were immediately sampled using the prescribed bacteria recovery methods. Following the sample recovery, the subjects’ hands were decontaminated with isopropyl alcohol, antibacterial soap, and topical antibiotic cream.

### Bacterial recovery method.

**(i) Bag method.** Plastic bags (nonsterile, low-density flat poly bag [1.5 mil] or equivalent; 29.2 cm × 31.8 cm) were placed on the subject’s right and left hands. Seventy-five ± 2-mL aliquots of stripping solution with neutralizer (0.075 M phosphate buffer with lecithin and Tween 20) were added to each bag. Each bag was secured at the wrist, and the hands were massaged for 1 min in a uniform manner. A 1-mL aliquot was obtained from the bagged hands within 1 min of completing the massage and placed into a 9-mL tube of Butterfield’s phosphate-buffered water with lecithin and Tween 80.

**(ii) Glove method.** Synthetic polyisoprene sterile surgical gloves (unlined, powder-free gloves with no antimicrobial properties in size extralarge) were pulled open by staff and placed on the subject’s right and left hands. Seventy-five ± 2-mL aliquots of stripping solution with neutralizer (0.075 M phosphate buffer with lecithin and Tween 20) were added to each glove. Each glove was secured at the wrist, and the hands were massaged for 1 min in a uniform manner. A 1-mL aliquot was obtained from the gloved hands within 1 min of completing the massage and placed into a 9-mL tube of Butterfield’s phosphate-buffered water with lecithin and Tween 80.

### Enumeration of bacteria from bags or gloves.

Serial 10-fold dilutions were performed with Butterfield’s phosphate-buffered water with lecithin and Tween 80 by using the initial 1-mL aliquot from the bagged or gloved hands. Dilution aliquots were plated onto TSA using standard spread plate-counting procedures. The plates were incubated at 20 to 25°C for 36 to 48 h to enhance pigmentation development. Colonies displaying red pigmentation typical of S. marcescens were counted. Plates yielding 25 to 250 colonies were counted using standard plate-counting procedures.

### Calculations and statistical analysis.

The number of bacteria per hand was calculated by multiplying the CFU per milliliter obtained in the plate count by 75, the volume in milliliters of stripping solution used in the bag. The CFU per hand was then converted to log_10_ counts and averaged, including the right and left hands. Then, the average and standard deviation for each recovery method (glove or bag) were calculated. The log_10_ bacterial counts from each recovery method were tested for normality using the Shapiro-Wilk and Anderson-Darling tests. Since the data were found to be not normally distributed, a nonparametric Wilcoxon signed-rank test was used to compare the recovery methods (bag versus glove). An analysis of variance (ANOVA) with α equal to 0.05 was used to assess other sources of data variation, including the collection day within the study and demographic categories, including sex, race, age, and hand size of subjects.
