# Supplementary material for: Comparative Recovery of Serratia marcescens Using Bags versus Gloves as Described in ASTM E1174-21 Health Care Personnel Handwash Method
Source: Microbiol Spectr. 2023 May 18;11(3):e01288-23. doi: 10.1128/spectrum.01288-23 (PMC10269534; doi:10.1128/spectrum.01288-23)

Supplemental Data For Publication

Comparative Recovery of *Serratia marcescens* using Bags vs Gloves as Described in ASTM E1174-21 Health Care Personnel Handwash Method

Table 1. Summary of demographic information for all subjects (N=46). The Henkel site completed with 24 subjects and the SGS site completed with 22 subjects.

	Total N (%)	Henkel	SGS
**Sex**			
Female	32 (70%)	18 (75%)	14 (64%)
Male	14 (30%)	6 (25%)	8 (36%)
**Race**			
American Indian or Alaska Native	2 (4%)	NA	2 (9%)
Asian	2 (4%)	1 (4%)	1 (5%)
Black or African American	11 (24%)	7 (29%)	4 (18%)
Native Hawaiian or other Pacific Islander	1 (2%)	NA	1 (5%)
White or Caucasian	30 (65%)	16 (67%)	14 (64%)
**Hand Size**			
Small	12 (26%)	7 (29%)	5 (23%)
Medium	19 (41%)	12 (50%)	7 (32%)
Large	13 (28%)	5 (21%)	8 (35%)
Extra Large	2 (4%)	NA	2 (9%)
**Age Group**			
18-40	7 (15%)	3 (13%)	4 (18%)
41-55	21 (46%)	11 (46%)	10 (45%)
56-65	18 (39%)	10 (42%)	8 (36%)

Figure 1. Photos of bag method (left) and glove (right) on the hand. Photos depict an extra-large hand without the 75 mL of stripping solution additionally added.

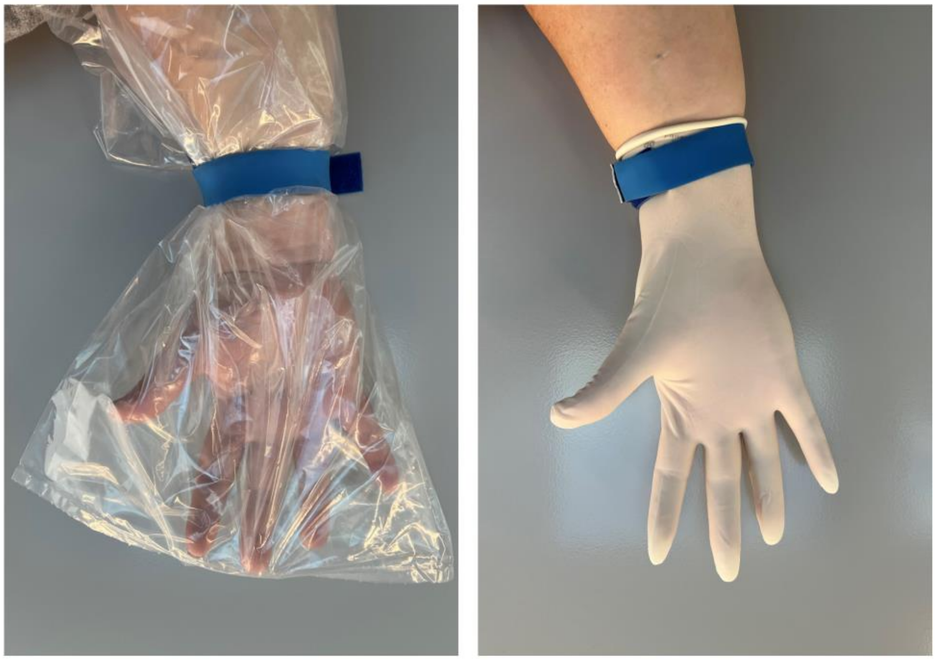

Supplement: Supplemental file 1 — Supplemental material. Download spectrum.01288-23-s0001.pdf, PDF file, 0.08 MB [file spectrum.01288-23-s0001.pdf]
